# Assessing the Accuracy of the Modified Chinese Autism Spectrum Rating Scale and Social Responsiveness Scale for Screening Autism Spectrum Disorder in Chinese Children

**DOI:** 10.1007/s12264-017-0114-5

**Published:** 2017-03-03

**Authors:** Bingrui Zhou, Hao Zhou, Lijie Wu, Xiaobing Zou, Xuerong Luo, Eric Fombonne, Yi Wang, Weili Yan, Xiu Xu

**Affiliations:** 1 Department of Child Healthcare, Children’s Hospital of Fudan University, Shanghai, 201102 China; 2 Department of Neurology, Children’s Hospital of Fudan University, Shanghai, 201102 China; 3 School of Public Health, Harbin Medical University, Harbin, 150081 China; 4 Child Development Center, The Third Affiliated Hospital, Sun Yat-Sen University, Guangzhou, 510630 China; 5 Department of Psychiatry, The Second Xiangya Hospital of Central South University, Changsha, 410011 China; 6 Oregon Health and Science University, Portland, OR 97239 USA; 7 Department of Clinical Epidemiology, Children’s Hospital of Fudan University, Shanghai, 201102 China

**Keywords:** Autism spectrum disorder, Screening accuracy, ROC analysis, Modified Chinese Autism Spectrum Rating Scale, Social Responsiveness Scale

## Abstract

The reported prevalence of autism spectrum disorder (ASD) has been increasing rapidly in many parts of the world. However, data on its prevalence in China are largely missing. Here, we assessed the suitability of the modified Chinese version of a newly-developed ASD screening tool, the Modified Chinese Autism Spectrum Rating Scales (MC-ASRS) in screening for ASD in Chinese children aged 6–12 years, through comparison with the Social Responsiveness Scale (SRS) that has been widely used for ASD screening. We recruited the parents/caregivers of 1588 typically-developing children and 190 children with ASD aged 6–12 years to complete the MC-ASRS and SRS, and evaluated the validity of both scales in discriminating children with ASD from those developing typically. The results showed that MC-ASRS performed as well as SRS in sensitivity, specificity, and area-under-the-curve (both >0.95) in receiver operating characteristic analysis, with a fair false-negative rate. These results suggest that MC-ASRS is a promising tool for screening for children with ASD in the general Chinese population.

## Introduction

In the past several decades, autism spectrum disorder (ASD) has become an increasingly important issue of concern worldwide. ASD consists of an array of disorders characterized by impairment in reciprocal social interaction and communication skills, and the presence of repetitive stereotypic behaviors/restricted interests [1, 2], with variability in symptom pattern, severity, associated cognitive and language ability, and prognosis [3]. Many studies have suggested that early identification, diagnosis, and intervention can ameliorate the prognosis of ASD [4–7].

The prevalence of ASD reported in various countries and regions has increased dramatically since 2000. Studies have suggested an estimated prevalence of ASD of ~1% in the general population [8]. In China, most reported epidemiological studies of ASD have been regional, with relatively small samples. Furthermore, the targeted clinical cases were variable, with most studies screening for children with classical autism, and some for those with ASD [9]. In addition, a lack of standard diagnostic instruments to assess the positively-screened individuals made the results less valid. The Autism Diagnostic Observation Schedule (ADOS) and Autism Diagnostic Interview-Revised (ADI-R) have generally been recommended for case confirmation in ASD epidemiology studies [10]. The shortcomings noted above make it difficult to directly compare the prevalence estimates from existing Chinese studies with those from recent studies in other parts of the world. With the support of a national program, we will conduct a multi-site epidemiological investigation of ASD in Chinese school-aged children (6–12 years old) using standard procedures of screening and diagnosis, making the prevalence comparable to existing results from developed countries. Above all, we needed to identify a screening instrument appropriate for our targeted population.

Currently in China, screening instruments available for 6–12-year-old children are mainly the Autism Behavior Checklist (ABC), Autism Spectrum Screening Questionnaire (ASSQ), and Social Communication Questionnaire (SCQ). There has been almost no research using the ABC in developed countries, making it difficult to make comparisons. Although a previous study showed good sensitivity and specificity of the ASSQ in differentiating children with ASD from healthy controls, as well as children with attention deficit/hyperactivity disorder (ADHD) and childhood-onset schizophrenia [11], the ASSQ was designed to identify children with high-functioning ASD, particularly Asperger syndrome. The positive rate using the ASSQ for level-1 screening may underestimate the prevalence of ASD. The SCQ is more commonly used in level-2 screening to discriminate ASD from other developmental disorders [12]. In recent years, the Social Responsiveness Scale has been introduced in the Taiwan region and shows good reliability and validity in Taiwan children [13].

The Autism Spectrum Rating Scale (ASRS) is a screening tool developed by Goldstein and Naglieri in 2009 [14], designed to measure autism-related behaviors in children and adolescents aged 2–18 years. The ASRS has full-length and short versions, both of which can be completed by parents or teachers. The full-length ASRS (6–18 years) consists of 3 scales: the ASRS scale for screening, the DSM-IV-TR scale for guiding diagnostic decisions, and the treatment scale for monitoring the effectiveness of intervention. The available age-range of the ASRS is large and appropriate for follow-up studies. In previous work, Zhou *et al.* [15, 16] translated the ASRS into Chinese and assayed its suitability for screening children with ASD from the general population. The results showed that the modified Chinese version of the ASRS, the MC-ASRS, shows good reliability and validity [16]. In the current study, we compared the screening accuracy of the MC-ASRS with that of the widely-used SRS in discriminating ASD cases in school-aged children, to further investigate the applicability of the MC-ASRS in first-level screening for ASD in China.

## Methods

### Study Design and Participants

The study was conducted from January to July, 2014, and enrolled children diagnosed with ASD according to DSM-V from both clinics and local autism rehabilitation centers, and typically-developing healthy children from communities, all aged 6–12 years. The children were recruited from Shanghai, Guangzhou, Harbin, and Changsha, representing four main areas of China, to ensure data quality and representativeness.

The ASD children were recruited from both clinics and local autism rehabilitation centers in the four cities. A clinical diagnosis of ASD was made according to the DSM-V criteria and confirmed by senior developmental pediatricians using the ADOS and ADI-R. Individuals were excluded if they were diagnosed with symptomatic autism (such as Rett syndrome and fragile X syndrome), inherited metabolic diseases, mental retardation caused by secondary brain injury, or psychiatric diseases such as schizophrenia and schizoaffective disorder.

Healthy, typically-developing age-matched children were recruited from communities in the 4 cities using convenient cluster sampling, to represent a healthy group without ASD. Parents of children with visual and/or auditory impairment and nervous system diseases were excluded.

## Instruments

### MC-ASRS (Full-Length Form, 6–18 Years, Parent Rating)

The original full-length ASRS (6–18 years) uses a five-point Likert scale ranging from ‘Never’ (0) to ‘Very Frequently’ (4) according to the frequency of the corresponding behavior, and has good psychometric properties [14]. It includes 71 items consisting of 3 scales, the ASRS scale for ASD screening, the DSM-IV-TR scale, and the treatment scale. In the ASRS scale, three subscales consisting of 60 items are used: Unusual Behaviors (UB, 24 items), Social/Communication (SC, 19 items), and Self-Regulation (SR, 17 items), the scores of which are raw scores. All raw scores are combined into a single composite score, the T-score [17]. A higher T-score indicates more obvious ASD features. The T-scores of ASRS follow a normal distribution with a normative mean of 50 and standard deviation of 10 [15], and the cut-off point is set to 60 (mean + 1 SD) [14].

With the permission of Goldstein and Naglieri and with approval by the Multi-Health System, our colleagues Zhou *et al*., in the team of the Research Special Fund for Public Welfare Industry of Health of China, translated the original ASRS into Chinese using a two-way procedure. They then found it to be a reliable and valid tool for screening ASD traits in general Chinese children, but its construct validity was not entirely satisfactory [15]. Therefore, they conducted exploratory factor analyses, after which they retained the original three-factor solution but excluded 12 items because of low factor loading (<0.3) or cross-loading, resulting in the Modified Chinese ASRS (MC-ASRS) that includes 59 items in the ASRS scale. The DSM-IV-TR and treatment scales of the MC-ASRS were retained from the original version. Then, confirmatory factor analyses for the MC-ASRS and the unmodified Chinese ASRS were performed in the same new Chinese sample. The results show that the model-fitting indices of the MC-ASRS are better than those of the unmodified version with the same cut-off T-score of 60, indicating that the MC-ASRS has better construct validity [16, 18].

### The Chinese Version of the Social Responsiveness Scale (Chinese SRS) - Parent

The original SRS was developed by Constantino and colleagues in 2002[19]. It consists of 65 items divided into 5 subscales: Social Awareness, Social Cognition, Social Communication, Social Motivation, and Autistic Mannerisms. It was designed to assess the social behavior of children aged 4–18 years. The SRS uses a four-point Likert-type questionnaire reported by the individual himself/herself or the caregiver according to the frequency of each behavior (“0” never to “3” always). A higher score indicates more severe social deficits and autistic behaviors. The raw SRS score can be converted to a T-score, but it is recommended to use the total raw score in research, in order to increase the comparability between studies [20]. Therefore, we used the raw SRS score in the current study. SRS scores are highly correlated with ADI-R scores (*r* = 0.65–0.77) [19]. The SRS performs well in psychometric properties across different cultures [13, 21, 22], including the Chinese version in the Taiwan region. The recommended cut-off for the raw in the Chinese version of SRS when used for screening for ASD in low-risk populations in China is 60 [23].

### Procedures

The research protocol was approved by the Research Ethics Committee of the Children’s Hospital of Fudan University ([2012] No. 185) before data collection. Parents of eligible children were invited to participate in the study and received a folder containing an informed consent letter, a general information sheet, the MC-ASRS and SRS scales, and guidance notes. Parents who signed the informed consent completed the two scales on two separate days with an intervening period of no more than two weeks. The order in which the MC-ASRS and SRS were assigned to the parents was done by simple randomization. For comparison with the SRS, only the parent version of the MC-ASRS was included in the analysis.

Data were entered after the questionnaires were retrieved from the sites by two separate groups of trained staff. Clinicians who administered the ADOS and ADI-R assessments were trained and certified.

### Statistical Analysis

We retrieved 1596 questionnaires from the general sample and 190 from the ASD sample. In the general sample, 3 individuals were excluded from analysis because both the MC-ASRS and SRS were ≥70, indicating high likelihood of ASD [24]. Indeed, further assessment using the ADOS and ADI-R confirmed that these children have ASD. In addition, 5 individuals in the clinical sample were excluded because they failed to complete both scales. Finally, the data from 1778 participants (1593 from the general population and 185 clinical ASD cases) were analyzed.

Descriptive statistics were computed for the scores on the selected instruments. Unpaired* t*-tests were used to compare means, and the* χ* ^2^ test was used to assess differences in proportions. First, differences between the scores in children with ASD and typically-developing children were investigated using independent sample* t*-tests when the distribution was robustly normal, or using the Mann-Whitney test when it was skewed. Then, using clinical diagnosis by DSM-V as the reference standard and the general sample as a typical control, we performed receiver-operator-characteristic (ROC) area-under-the-curve (AUC) analyses using the same cut-off score of 60, to assess and compare the screening accuracy of the MC-ASRS and the SRS. Based on these results, we further calculated and compared the sensitivities, specificities, positive and negative predictive values, and positive and negative likelihood ratios for the MC-ASRS and SRS. Sensitivity was calculated as the percentage of children with ASD who tested positive, while specificity was calculated as the percentage of children without ASD who tested negative. The positive predictive value (PPV) was determined as the percentage of all children testing positive who were later diagnosed with ASD, while the negative predictive value (NPV) was the percentage of all children testing negative who did not have ASD. The 95% confidence interval (95% CI) was computed by the Wilson method. Stata SE 11.0 was used to conduct the statistical analyses.

## Results

The demographic characteristics of the two samples are shown in Table 1. The mean age of all participants was 8.8 years (SD = 1.8), and those of the clinical ASD cases and general sample showed no significant differences (8.9 ± 1.8 *vs* 8.4 ± 1.9, *P* = 0.178). The sex ratio of the clinical group was 7.26:1 (male:female), while that of the general sample was 1.05:1. The percentages of participants from each site showed no significant differences.

**Table 1:** Demographic characteristics of the general sample and ASD cases.

	General sample			ASD cases		
	*n*	*χ* ^2^	*P*	*n*	*χ* ^2^	*P*
City (%)	1596			190		
1	415			57		
2	412			41		
3	345			42		
4	424			50		
Sex (% male)	816 (51.13%)			167 (87.89%)		
6 years	82 (53.95%)	4.3697	0.627	35 (89.74%)	2.7481	0.840
7 years	158 (51.97%)			31 (86.11%)		
8 years	128 (51.82%)			24 (88.89%)		
9 years	153 (52.40%)			27 (90.00%)		
10 years	107 (45.34%)			18 (85.71%)		
11 years	125 (50.40%)			23 (92.00%)		
12 years	63 (53.85%)			9 (75.00%)		
Administrator (%)	1561			179		
Father	510 (32.67%)			33 (18.44%)		
Mother	1010 (64.70%)			125 (69.83%)		
Grandfather	17 (1.09%)			5 (2.79%)		
Grandmother	14 (0.90%)			14 (7.82%)		
Other	10 (0.64%)			2 (1.12%)		

As expected, the clinical sample scored significantly higher than the community sample on both the MC-ASRS and the SRS (both *P* <0.001, Table 2).

**Table 2:** MC-ASRS and SRS scores in the general sample and ASD cases.

Scales	General sample (*n* = 1593)	ASD cases (*n* = 185)	Effect size*	*t* value	*P*
ASRS scale					
T-Score	47.89 ± 7.85	67.06 ± 8.73	−19.17 ± 0.62	−31.07	<0.001
SC	24.30 ± 11.95	50.01 ± 13.54	−25.72±0.94	−27.29	<0.001
UB	27.74 ± 10.79	46.84 ± 13.52	−19.10±0.86	−22.15	<0.001
SR	16.91 ± 7.52	29.46 ± 9.10	−12.54±0.60	−20.99	<0.001
DSM-IV-TR scale	41.86 ± 13.07	74.80 ± 14.91	−32.94 ± 1.03	−27.72	<0.001
SRS score	43.15 ± 18.22	103.33 ± 25.70	−60.17 ± 1.49	−40.49	<0.001

*MC-ASRS* Modified Chinese Autism Spectrum Rating Scales, *SC* Social Communication, *SR* Self Regulation, *SRS* Social Responsiveness Scale, *UB* Unusual Behavior.

* Difference ± SE. The SC, UB, SR, DSM-IV-TR, and SRS scores are raw, and the T-score of MC-ASRS is composite.

In general, with the same cut-off point of 60, the MC-ASRS and SRS performed similarly in screening for ASD cases in the general sample. The sensitivity of MC-ASRS was a little lower than SRS (MC-ASRS vs SRS, 93% *vs* 96.8%), while the specificity was in the opposite direction (83.2% *vs* 82.2%). The NPVs were both very high (≥99%), suggesting that it was very unlikely that a child scoring <60 would be diagnosed as having ASD, while the PPVs were relatively low, indicating high false-positive rates of both instruments. The positive likelihood ratios were similar, but the negative likelihood ratio of SRS was lower than that of MC-ASRS (Table 3).

**Table 3:** Comparison of diagnostic accuracy between the MC-ASRS and SRS scales in screening for autism spectrum disorder in children aged 6–12 years.

	MC-ASRS	SRS
True positives	172	179
False negatives	13	6
False positives	268	284
True negatives	1325	1309
Sensitivity (95% CI)	93.0% (88.3–96.2)	96.8% (93.1–98.8)
Specificity (95% CI)	83.2% (81.2–85.1)	82.2% (80.2–84.0)
Positive likelihood ratio (95% CI)	553% (492–621)	543% (487–605)
Negative likelihood ratio (95% CI)	8.45%(5.0–14.3)	3.95% (1.8–8.7)
Odds ratio (95% CI)	65.4(36.9–116.0)	138.0 (61.6–307.0)
Positive predictive value (95% CI)	39.1% (34.5–43.8)	38.7% (34.2–43.3)
Negative predictive value (95% CI)	99.0% (98.3–99.5)	99.5% (99.0–99.8)
False-positive rate, %	16.8%	17.8%

*MC-ASRS* Modified Chinese Autism Spectrum Rating Scales, *SRS* Social Responsiveness Scale, *95% CI* 95% confidence interval.

**Fig. 1:**
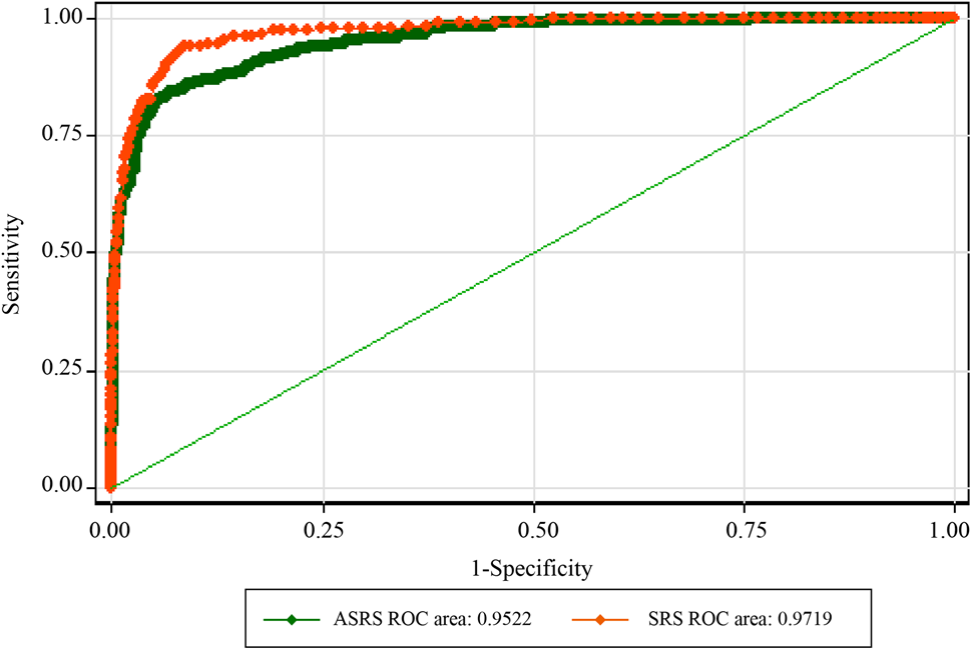
ROC curves and AUCs of MC-ASRS T-scores and total raw SRS scores. *AUC* area-under-the-curve, *ROC* receiver operating characteristic, *MC-ASRS* Modified Chinese Autism Spectrum Rating Scales, *SRS* Social Responsiveness Scale.

The performance of the MC-ASRS and SRS were compared mainly through the AUCs under ROC curves. Both performed well in distinguishing ASD cases from typically-developing children (both AUCs >0.95), SRS being slightly better than MC-ASRS (MC-ASRS 0.9522 *vs* SRS 0.9719, *P* = 0.0011) (Fig. 1).

At all four sites, parents were the main administrators, but the proportion of mothers administering the scales was higher than that of fathers. In considering the possible discrepancy resulting from the father or the mother administering the scales, we separately calculated and compared the AUCs of the ROC curves for the two subsamples. The results showed no significant differences in the AUC of the MC-ASRS between the fathers or the mothers administering the questionnaire, with a higher AUC for the mothers completing the SRS than the fathers (Table 4).

**Table 4:** AUCs of ROCs of the MC-ASRS and SRS for main administrators.

Scales	Administrator	*n*	AUC	SE	95% CI	*χ* ^*2*^	*P*
MC-ASRS	Father	543	0.9502	0.0199	0.9111–0.9892	0.11	0.8021
	Mother	1135	0.9506	0.0099	0.9312–0.9698		
SRS	Father	543	0.9435	0.0217	0.9010–0.9859	15.24	0.0001
	Mother	1135	0.9777	0.0054	0.9671–0.9883		

*AUC* area-under-the-curve, *MC-ASRS* Modified Chinese Autism Spectrum Rating Scales, *ROC* receiver operating characteristic, *SRS* Social Responsiveness Scale.

## Discussion

### Screening Accuracy of the MC-ASRS and SRS

In a previous study, the original ASRS was translated into Chinese [15] and modified based on the results of exploratory and confirmatory factor analysis to achieve a better construct validity for the Chinese population aged 6–12 years [16]. Also, the norm of the MC-ASRS in 6–12-year-old Chinese children was established [25]. Here, we investigated the screening accuracy of the MC-ASRS in a multicenter study, by comparing it with the SRS, a widely-used tool for ASD screening. Our results showed that the MC-ASRS effectively identified children diagnosed with ASD using the DSM-V criteria, with screening accuracy similar to that of the SRS. When separated into two subsamples of administrators (father and mother), the results of both instruments were still both excellent.

As a newly-developed scale, the screening accuracy of the ASRS has not been systematically examined. We found good to excellent sensitivity and specificity for the cut-off T-score of 60 on the parent report. As to the parent-reported SRS, the estimate of sensitivity with regard to ASD classification according the DSM-V criteria was similar to that of a previous study [23], using samples with only typically-developing children and children with ASD.

Predictive values depend upon the prevalence of the targeted disease [26], while the likelihood ratios are relatively independent and also more steady when used in evaluating screening accuracy. In the current study, the NPVs of the two instruments were similarly high, while the PPVs were almost equally low, partly because of the low prevalence of ASD classification in our sample (185/1778, 10.4%). It was noted that the NLR of the MC-ASRS was higher than that of the SRS, indicating that the MC-ASRS has slightly greater but still acceptable potential [27] to misjudge an ASD case for a typical child than the SRS.

### Characteristics of the Two Scales

The parent version of the SRS is a widely-used scale designed to evaluate the social ability of children in the general population for screening purposes. There have been many diagnostic validity studies of the SRS in different countries [12, 13, 21, 28–31]. In the USA, German, and Chinese studies, the total SRS score performs well in differentiating children with ASD from typically-developing children. Our study concurred with these results. However, studies, including the original validation study [19], have also suggested that the SRS has lower screening accuracy in a complicated group of other mental disorders (such as intellectual disability, language disorder, ADHD, and ODD/CD), especially in children with a lower IQ and with greater behavioral problems [12]. The reason could be great overlap of communication and social interaction symptoms in children with ASD and other mental disorders and insufficient items focused on repetitive and restricted behaviors (RRBs) – another pivotal and characteristic domain of ASD – in the SRS. The majority of items (53/65) in the SRS describe normal or abnormal responses in social situations, focusing on the severity of the social communication deficit, while 12 items describe autistic mannerisms. The score generated by the SRS is an index of impairments in reciprocal social behaviors; some items are even geared toward other domains focusing on social aspects [32]. Furthermore, several items are descriptive of common symptoms of ASD, as well as of other neuropsychiatric disorders. Therefore, some disorders with social impairment showed overlapping SRS scores and could not be efficaciously differentiated from ASD [33].

The ASRS is a relatively new screening tool specifically for autistic traits. The scales include items related to the comprehensive symptoms and associated behaviors of ASD, including Asperger’s Syndrome and Pervasive Developmental Disorder-Not Otherwise Specified (PDD-NOS). The structure of the scales is consistent with the 3 symptomatic domains of the criteria, and all domains are covered in significant proportions. Even in the UB subscale, there are three key areas about RRBs: language stereotypes, behavioral rigidity, and sensory sensitivity, which could improve the ability to discriminate between children with ASD and children with other psychiatric disorders. The original ASRS study showed that the scores on the ASRS can effectively distinguish individuals with ASD from typically-developing individuals and those with other diagnoses [17]. However, further research is necessary in different countries and cultures to assess how the ASRS performs when differentiating children with ASD from those with other developmental neurological disorders.

### Strengths, Limitations, and Prospects

To our knowledge, the current study is the first to explore the screening accuracy of the MC-ASRS, and compare it with the SRS, another well-established ASD screening scale. The strengths of our study include a relatively large sample size and wide age-range, which could enhance the validity of the scales in subsequent studies. One limitation of this study is that the sample did not include children with other diagnoses, especially other developmental neurological disorders.

The screening accuracy of the two instruments may have been overestimated since children in the case group had been previously diagnosed and received special education, resulting in their parents’ or caregivers’ having a better understanding of the disorder and responding well to the items on the questionnaires, as compared to parents without previous knowledge of ASD. Therefore, when using the ASRS and SRS to screen for autistic traits in the general population, caution should be exercised. In future studies, it would be better to recruit the clinical subjects and complete the questionnaires during the first visit to reduce bias. In summary, the MC-ASRS shows good performance in screening for children with ASD in the general Chinese population aged 6–12 years, with effectiveness similar to the SRS. Further larger-scale and more sophisticated studies are needed to determine its suitability in screening children with ASD from those with other developmental neurological disorders.
